# Supplementary material for: Developing objective tools to study rock hyrax (Procavia capensis) behaviour in the field
Source: PLoS One. 2026 Feb 23;21(2):e0343302. doi: 10.1371/journal.pone.0343302 (PMC12928569; doi:10.1371/journal.pone.0343302)
Supplement: S1 File — Cluster assignment of all the constellations, R2 of each constellation with its own cluster, and with the next best cluster are listed on the left side of the map. (ZIP) [file pone.0343302.s001.zip › SI/Tables.docx]

**Supplementary materials**

**Table SI1: Publications that include rock hyrax behaviour**

| **Number** | **Year published** | **Authors** | **Title** | **Behaviours studied** |
| --- | --- | --- | --- | --- |
| 1 | 1970 | Sale, J. B. | The behaviour of the resting rock hyrax in relation to its environment. | feed, fight, rest, suckle, play, heap, huddle, solitary rest, bask |
| 2 | 1977 | Fourie, L. J. | Acoustic communication in the rock hyrax, *Procavia capensis*. | grunt, growl, snarl, spit, wail, wail-bark, yelp, rasp, hiccup, cough, hoarse moan, coo, whine, squeak, yap, sharp bark, repetitious bark, squeal, whistle, whistle chirrup, harsh chirrup, harsh twitter, soft twitter, snort, pant, sneeze, teeth gnash |
| 3 | 1982 | Hoeck, H. N., Klein, H., & Hoeck, P. | Flexible social organization in hyrax. | chase, flight, sniff, bite, huddled, fight, copulate, territorial call, jump, dorsal hair erect, guard, threat, mount |
| 4 | 1983 | Fourie, L. J. | The population dynamics of the rock hyrax *Procavia capensis* (Pallas, 1766) in the Mountain Zebra National Park. | groom, browse, sit, twisted, prostrate upright, Lay, sandbathe, bask, play, chase, nurse, mate, pilo-erection of hair around the dorsal gland, chase, bite, close bodily contact, approach, sniff, present hind-quarters, mount, grunt, copulate, scratch, guard, alarm call, freeze, squeak |
| 5 | 1985 | Caro, T. M., & Alawi, R. M. | Comparative aspects of behavioural development in two species of free-living hyrax. | fight, chase, bite, mount, play |
| 6 | 1987 | Fourie, L. J. & Perrin, M | Social behaviour and spatial relationships of the rock hyrax. | nurse, suckle, bask, social play, mate, chase, growl, flare of dorsal gland hairs, bite, sniff, copulation, infanticide |
| 7 | 1989 | Hoeck, H. N. | Demography and competition in hyrax: a 17 years study. | graze, browse, breed |
| 8 | 2002 | Barry, R. E., & Mundy, P. J. | Seasonal variation in the degree of heterospecific association of two syntopic hyraxes (*Heterohyrax brucei* and *Procavia capensis*) exhibiting synchronous parturition. | bask, nurse, vigilance, mate |
| 9 | 2008 | Koren, L., Mokady, O., & Geffen, E. | Social status and cortisol levels in singing rock hyraxes. | sing, mate |
| 10 | 2009 | Koren, L., & Geffen, E | Androgens and social status in female rock hyraxes. | approach, bite, push, run, retreat |
| 11 | 2009 | Koren, L., & Geffen, E | Complex call in male rock hyrax (*Procavia capensis*): a multi-information distributing channel. | approach, bite, push, flee, retreat, sing, mate |
| 12 | 2011 | Fanson, K. V., Fanson, B. G., & Brown, J. S. | Using path analysis to explore vigilance behavior in the rock hyrax (*Procavia capensis*). | forage, vigilance, alert, alarm call |
| 13 | 2011 | Ilany, A., Barocas, A., Koren, L., Kam, M., & Geffen, E. | Do singing rock hyraxes exploit conspecific calls to gain attention? | play, sing, pup scream |
| 14 | 2011 | Koren, L., & Geffen, E. | Individual identity is communicated through multiple pathways in male rock hyrax (*Procavia capensis*) songs. | sing, mate |
| 15 | 2012 | Kershenbaum, A., Ilany, A., Blaustein, L., & Geffen, E | Syntactic structure and geographical dialects in the songs of male rock hyraxes. | sing |
| 16 | 2013 | Ilany, A., Barocas, A., Kam, M., Ilany, T., & Geffen, E. | The energy cost of singing in wild rock hyrax males: evidence for an index signal. | growl, grind molars, snap, chase, bite, sing, forage, grunt, howl sound, twitter, click |
| 17 | 2014 | Demartsev, V., Kershenbaum, A., Ilany, A., Barocas, A., Bar Ziv, E., Koren, L., & Geffen, E. | Male hyraxes increase song complexity and duration in the presence of alert individuals. | sing, fight, alarm call, pup scream, mate |
| 18 | 2015 | Naylor, A. J. | The occurrence, behaviour and public perception of rock hyraxes, *Procavia capensis*, in urban areas. | bask, sunbath, vigilance, travel, feed, forage, groom, in refuge, chase, allogroom, sniff, huddle, heap, play |
| 19 | 2016 | Bar Ziv, E., Ilany, A., Demartsev, V., Barocas, A., Geffen, E., & Koren, L. | Individual, social, and sexual niche traits affect copulation success in a polygynandrous mating system. | copulation, mate-guarded, courtship, mount, rejection, interference |
| 20 | 2016 | Demartsev, V., Bar Ziv, E., Shani, U., Goll, Y., Koren, L., & Geffen, E. | Harsh vocal elements affect counter-singing dynamics in male rock hyrax. | sing |
| 21 | 2016 | Demartsev, V., Ilany, A., Barocas, A., Bar Ziv, E., Schnitzer, I., Koren, L., & Geffen, E. | A mixed strategy of counter-singing behavior in male rock hyrax vocal competitions. | sing |
| 22 | 2016 | Lake, E. | The effect of predator activity, weather and habitat variation on activity patterns of rock and bush hyrax (*Procavia capensis* and *Heterohyrax brucei*) in a mountainous environment. | forage, browse, graze, bask, fight, rest, travel, socialise |
| 23 | 2017 | Demartsev, V., Ilany, A., Kershenbaum, A., Geva, Y., Margalit, O., Schnitzer, I., Barocas, A., Einat, B., Koren, L., & Geffen, E. | The progression pattern of male hyrax songs and the role of climactic ending. | sing, mate-guard |
| 24 | 2017 | Goll, Y., Demartsev, V., Koren, L., & Geffen, E. | Male hyraxes increase countersinging as strangers become ‘nasty neighbours’ | sing |
| 25 | 2019 | Demartsev, V., Gordon, N., Barocas, A., Bar-Ziv, E., Ilany, T., Goll, Y., Ilany, A., & Geffen, E. | The “Law of Brevity” in animal communication: Sex-specific signaling optimization is determined by call amplitude rather than duration | twitter, growl, howl, click, squeek, bark, wail-bark, grunt, whine, trill, coo, sing |
| 26 | 2019 | Koren, L., Weissman, Y., Schnitzer, I., Beukeboom, R., Bar Ziv, E., Demartsev, V., Barocas, A., Ilany, A., & Geffen, E. | Sexually opposite effects of testosterone on mating success in wild rock hyrax. | mate, mate guard, copulation, mount, rejection, approach, retreat |
| 27 | 2019 | Weissman, Y. A., Demartsev, V., Ilany, A., Barocas, A., Bar-Ziv, E., Geffen, E., & Koren, L. | Social context mediates testosterone's effect on snort acoustics in male hyrax songs | sing, alarm call |
| 28 | 2019 | Weissman, Y. A., Demartsev, V., Ilany, A., Barocas, A., Bar-Ziv, E., Shnitzer, I., Geffen, E., & Koren, L. | Acoustic stability in hyrax snorts: vocal tightrope-walkers or wrathful verbal assailants? | sing, browse, alarm call |
| 29 | 2020 | Weissman, Y. A., Demartsev, V., Ilany, A., Barocas, A., Bar-Ziv, E., Koren, L., & Geffen, E. | A crescendo in the inner structure of snorts: a reflection of increasing arousal in rock hyrax songs? | sing, pup scream |
| 30 | 2022 | Goll, Y., Bordes, C., Weissman, Y. A., Shnitzer, I., Beukeboom, R., Ilany, A., Koren, L., & Geffen, E. | Sex-associated and context-dependent leadership in the rock hyrax. | run, rest, feed, mate-guard, mate |
| 31 | 2022 | Hemelrijk, C. K., Seex, L., Pederboni, M., Ilany, A., Geffen, E., & Koren, L. | Adult sex ratios and partial dominance of females over males in the rock hyrax. | fight, aggression, copulate, rest, attack, chase, flee, displace, retreat, threat, kill |
| 32 | 2023 | Demartsev, V., Haddas‐Sasson, M., Ilany, A., Koren, L., & Geffen, E. | Male rock hyraxes that maintain an isochronous song rhythm achieve higher reproductive success | sing, forage, mate, babysit |
| 33 | 2023 | Frydman, G., Goll, Y., Geffen, E., & Koren, L. | Sex differences in frequencies in a species with modest sexual size dimorphism | sing, trill, growl, howl, squeak, twitter |
| 34 | 2023 | Goll, Y., Bordes, C., Weissman, Y. A., Shnitzer, I., Beukeboom, R., Ilany, A., Koren, L., & Geffen, E. | The interaction between cortisol and testosterone predicts leadership within rock hyrax social groups | emerge, approach, run, feed, sleep, mate, copulate, pup scream, alarm call |

1. Sale, J. B. (1970). The behaviour of the resting rock hyrax in relation to its environment. African Zoology, 5(1).

2.Fourie, P. B. (1977). Acoustic communication in the rock hyrax, *Procavia capensis*. Zeitschrift für Tierpsychologie, 44(2), 194-219.

3.Hoeck, H. N., Klein, H., & Hoeck, P. (1982). Flexible Social Organization in hyrax 1. Zeitschrift für Tierpsychologie, 59(4), 265-298.

4.Fourie, L. J. (1983). The population dynamics of the rock hyrax *Procavia capensis* (Pallas, 1766) in the Mountain Zebra national park (Doctoral dissertation, Rhodes University).

5.Caro, T. M., & Alawi, R. M. (1985). Comparative aspects of behavioural development in two species of free-living hyrax. Behaviour, 95(1-2), 87-109.

6.Fourie, L.J. & Perrin, M. (1987). Social behaviour and spatial relationships of the rock hyrax. South African Journal of Wildlife Research-24-month delayed open access, 17(3), 91-98.

7.Hoeck, H. N. (1989). Demography and competition in hyrax: a 17-year study. Oecologia, 79, 353-360.

8. Barry, R. E., & Mundy, P. J. (2002). Seasonal variation in the degree of heterospecific association of two syntopic hyraxes (*Heterohyrax brucei* and *Procavia capensis*) exhibiting synchronous parturition. *Behavioral Ecology and Sociobiology*, *52*, 177-181.

9.Koren, L., Mokady, O., & Geffen, E. (2008). Social status and cortisol levels in singing rock hyraxes. Hormones and behavior, 54(1), 212-216.

10.Koren, L., & Geffen, E. (2009). Androgens and social status in female rock hyraxes. Animal Behaviour, 77(1), 233-238.

11.Koren, L., & Geffen, E. (2009). Complex call in male rock hyrax (Procavia capensis): a multi-information distributing channel. Behavioral Ecology and Sociobiology, 63, 581-590.

12.Fanson, K. V., Fanson, B. G., & Brown, J. S. (2011). Using path analysis to explore vigilance behavior in the rock hyrax (*Procavia capensis*). Journal of mammalogy, 92(1), 78-85.

13.Ilany, A., Barocas, A., Koren, L., Kam, M., & Geffen, E. (2011). Do singing rock hyraxes exploit conspecific calls to gain attention? PLoS One, 6(12), e28612.

14.Koren, L., & Geffen, E. (2011). Individual identity is communicated through multiple pathways in male rock hyrax (*Procavia capensis*) songs. Behavioral Ecology and Sociobiology, 65, 675-684.

15.Kershenbaum, A., Ilany, A., Blaustein, L., & Geffen, E. (2012). Syntactic structure and geographical dialects in the songs of male rock hyraxes. Proceedings of the Royal Society B: Biological Sciences, 279(1740), 2974-2981.

16.Ilany, A., Barocas, A., Kam, M., Ilany, T., & Geffen, E. (2013). The energy cost of singing in wild rock hyrax males: evidence for an index signal. Animal Behaviour, 85(5), 995-1001.

17.Demartsev, V., Kershenbaum, A., Ilany, A., Barocas, A., Bar Ziv, E., Koren, L., & Geffen, E. (2014). Male hyraxes increase song complexity and duration in the presence of alert individuals. Behavioral Ecology, 25(6), 1451-1458.

18.Naylor, A. J. (2015). The occurrence, behaviour and public perception of rock hyraxes, *Procavia capensis*, in urban areas. University of the Witwatersrand, Johannesburg (South Africa).

19.Bar Ziv, E., Ilany, A., Demartsev, V., Barocas, A., Geffen, E., & Koren, L. (2016). Individual, social, and sexual niche traits affect copulation success in a polygynandrous mating system. Behavioral Ecology and Sociobiology, 70, 901-912.

20.Demartsev, V., Bar Ziv, E., Shani, U., Goll, Y., Koren, L., & Geffen, E. (2016). Harsh vocal elements affect counter-singing dynamics in male rock hyrax. Behavioral Ecology, 27(5), 1397-1404.

21.Demartsev, V., Ilany, A., Barocas, A., Bar Ziv, E., Schnitzer, I., Koren, L., & Geffen, E. (2016). A mixed strategy of counter-singing behavior in male rock hyrax vocal competitions. Behavioral ecology and sociobiology, 70, 2185-2193.

22.Lake, E. (2016). The effect of predator activity, weather and habitat variation on activity patterns of rock and bush hyrax (*Procavia capensis* and *Heterohyrax brucei*) in a mountainous environment (Doctoral dissertation, Durham University).

23.Demartsev, V., Ilany, A., Kershenbaum, A., Geva, Y., Margalit, O., Schnitzer, I., Barocas, A., Einat, B., Koren, L., & Geffen, E. (2017). The progression pattern of male hyrax songs and the role of climactic ending. Scientific Reports, 7(1), 2794.

24.Goll, Y., Demartsev, V., Koren, L., & Geffen, E. (2017). Male hyraxes increase countersinging as strangers become ‘nasty neighbours’. Animal Behaviour, 134, 9-14.

25.Demartsev, V., Gordon, N., Barocas, A., Bar-Ziv, E., Ilany, T., Goll, Y., Ilany, A., & Geffen, E. (2019). The “Law of Brevity” in animal communication: Sex-specific signaling optimization is determined by call amplitude rather than duration. Evolution letters, 3(6), 623-634.

26.Koren, L., Weissman, Y., Schnitzer, I., Beukeboom, R., Bar Ziv, E., Demartsev, V., Barocas, A., Ilany, A., & Geffen, E. (2019). Sexually opposite effects of testosterone on mating success in wild rock hyrax. Behavioral Ecology, 30(6), 1611-1617.

27.Weissman, Y. A., Demartsev, V., Ilany, A., Barocas, A., Bar-Ziv, E., Geffen, E., & Koren, L. (2019). Social context mediates testosterone's effect on snort acoustics in male hyrax songs. Hormones and Behavior, 114, 104535.

28.Weissman, Y. A., Demartsev, V., Ilany, A., Barocas, A., Bar-Ziv, E., Shnitzer, I., Geffen, E., & Koren, L. (2019). Acoustic stability in hyrax snorts: vocal tightrope-walkers or wrathful verbal assailants? Behavioral Ecology, 30(1), 223-230.

29.Weissman, Y. A., Demartsev, V., Ilany, A., Barocas, A., Bar-Ziv, E., Koren, L., & Geffen, E. (2020). A crescendo in the inner structure of snorts: a reflection of increasing arousal in rock hyrax songs? Animal Behaviour, 166, 163-170.

30.Goll, Y., Bordes, C., Weissman, Y. A., Shnitzer, I., Beukeboom, R., Ilany, A., Koren, L., & Geffen, E. (2022). Sex-associated and context-dependent leadership in the rock hyrax. Iscience, 25(4).

31.Hemelrijk, C. K., Seex, L., Pederboni, M., Ilany, A., Geffen, E., & Koren, L. (2022). Adult sex ratios and partial dominance of females over males in the rock hyrax. Frontiers in Ecology and Evolution, 10, 1004919.

32.Demartsev, V., Haddas‐Sasson, M., Ilany, A., Koren, L., & Geffen, E. (2023). Male rock hyraxes that maintain an isochronous song rhythm achieve higher reproductive success. Journal of Animal Ecology, 92(8), 1520-1531.

33.Frydman, G., Goll, Y., Geffen, E., & Koren, L. (2023). Sex differences in frequencies in a species with modest sexual size dimorphism. Bioacoustics, 32(2), 230-240.

34.Goll, Y., Bordes, C., Weissman, Y. A., Shnitzer, I., Beukeboom, R., Ilany, A., Koren, L., & Geffen, E. (2023). The interaction between cortisol and testosterone predicts leadership within rock hyrax social groups. Scientific Reports, 13(1), 14857.

**Table SI2: Groups observed during the 2024 field season.**

| **Group name** | **Number of adult females** | **Number of adult males** | **Number of juvenile females** | **Number of juvenile males** |
| --- | --- | --- | --- | --- |
| **Suckot** | 4 | 0 | 1 | 0 |
| **Isiim** | 8 | 1 | 3 | 1 |
| **Mountain** | 2 | 1 | 1 | 4 |
| **David** | 6 | 1 | 5 | 3 |
