# Supplementary material for: Developing objective tools to study rock hyrax (Procavia capensis) behaviour in the field
Source: PLoS One. 2026 Feb 23;21(2):e0343302. doi: 10.1371/journal.pone.0343302 (PMC12928569; doi:10.1371/journal.pone.0343302)
Supplement: S1 File — Cluster assignment of all the constellations, R2 of each constellation with its own cluster, and with the next best cluster are listed on the left side of the map. (ZIP) [file pone.0343302.s001.zip › SI/Fig. S1.pdf]

Cluster	Members	R^2 own cluster	R^2 next cluster
1	copulate	0.941	0.172
1	mount	0.941	0.172
1	present	0.788	0.212
1	mate rejection	0.736	0.041
1	back to back	0.214	0.044
2	fight	0.898	0.007
2	bite	0.874	0.034
2	chase	0.193	0.015
3	babysit	0.642	0.032
3	play	0.391	0.020
3	nurse	0.330	0.020
3	coprophagy	0.159	0.020
4	graze	0.404	0.011
4	join	0.310	0.021
4	follow	0.271	0.004
4	browse	0.262	0.032
5	approach	0.615	0.236
5	sniff	0.494	0.044
5	alarm call	0.183	0.010
6	displacement	0.557	0.009
6	sing	0.557	0.027
7	pup screams	0.509	0.004
7	social touch	0.509	0.008

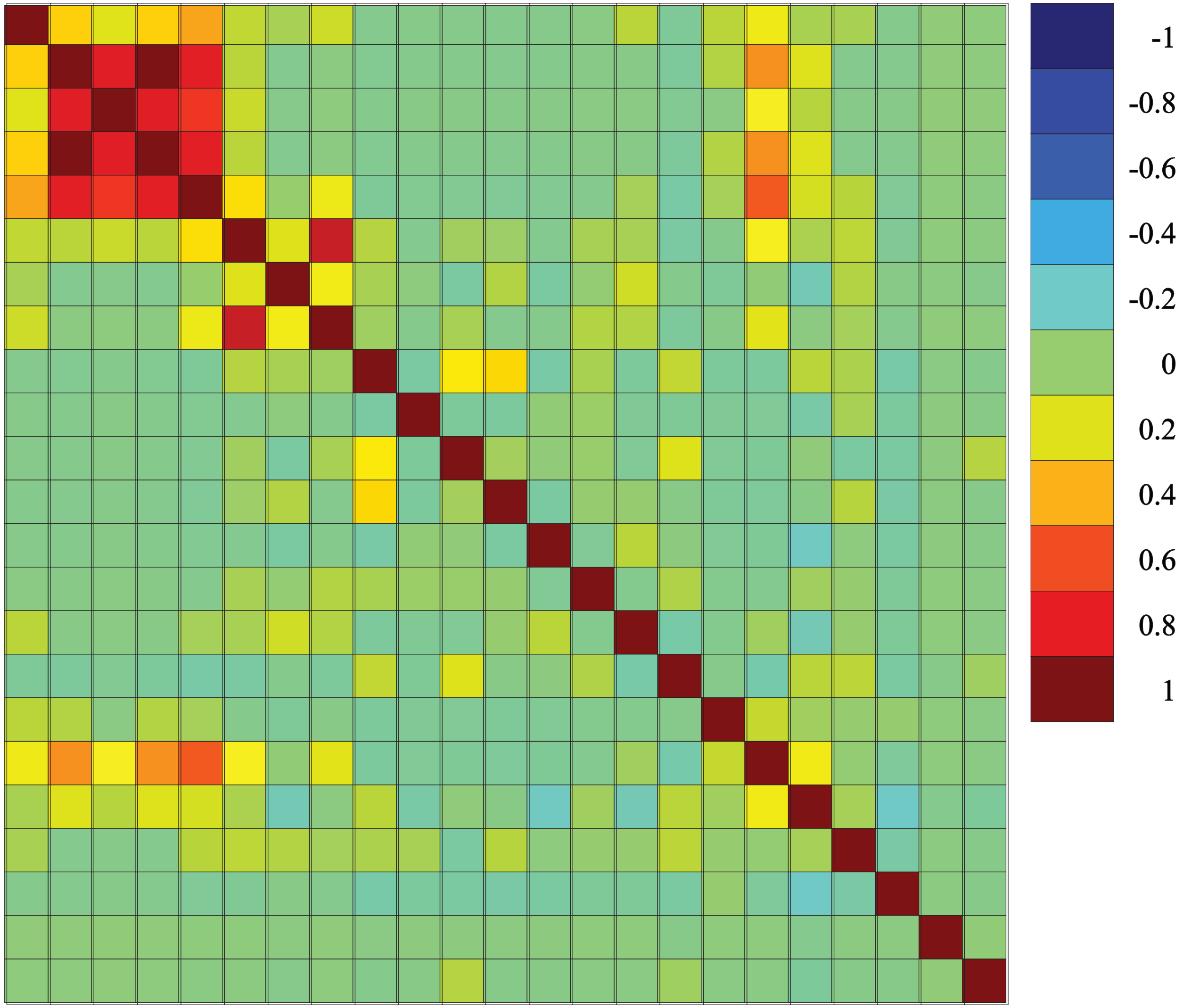

Fig. S1: Cluster Variables (SAS PROC VARCLUS) model results. Correlation color map for the 23 constellations. Cluster assignment of all the constellations, R^2 of each constellation with its own cluster, and with the next best cluster are listed on the left side of the map.
